# Rupture of hepatic hemangioma with hemoperitoneum due to spontaneous gallbladder perforation: A unique case report

**DOI:** 10.1097/MD.0000000000006110

**Published:** 2017-02-24

**Authors:** Qing-Hong Ke, Chun-Jun Zhang, Hai-Feng Huang

**Affiliations:** a Department of Hepatobiliary and Pancreatic Surgery, The First Affiliated Hospital, College of Medicine, Zhejiang University, Hangzhou; b Department of Hepatobiliary and Pancreatic Surgery, Shengzhou Branch of The First Affiliated Hospital, College of Medicine, Zhejiang University, Shengzhou, Zhejiang, P.R. China.

**Keywords:** enucleation, gallbladder perforation, hemoperitoneum, hepatic hemangioma, rupture

## Abstract

**Introduction:**

Hemangiomas are common benign tumors of the liver. Spontaneous rupture is a rare complication, occurring most commonly in giant hemangiomas. Rupture of a hemangioma with hemoperitoneum is a serious development and can be fatal if not managed promptly.

The present study reports the unique case of a man who experienced rupture and hemorrhage of a hepatic hemangioma (HH) due to perforation of the gallbladder fundus. After en block resection of the hemangioma and gallbladder using the Pringle maneuver, the patient made an uneventful recovery without complications.

To our knowledge, spontaneous rupture of HH secondary to gallbladder perforation has not been reported in the literature. This case highlights a unique, rare cause of ruptured HH and the need to consider appropriate treatment for some hemangiomas to avoid this potentially fatal complication.

**Conclusion:**

The current case may provide additional support for treatment of HH due to the potential for spontaneous rupture. For patients with ruptured HH, enucleation with the Pringle maneuver is recommended.

## 1 Introduction

Hemangioma is the most common solid benign tumor of the liver.^[1,2]^ Spontaneous rupture in hepatic hemangiomas (HH) is rare and seems to be related to the size of the lesion.^[3–5]^ If the patient receives steroid therapy, the risk of rupture increases further.^[3]^ Spontaneous rupture of HH is considered a life-threatening situation. Thus, appropriate treatment of HH must be determined to avoid this complication. We presented an extremely uncommon case of spontaneous rupture of HH where perforation of the gallbladder caused the tumor capsule to tear. En block resection of the hemangioma and gallbladder was performed using the Pringle maneuver. The patient recovered without complications.

## 2 Case report

This study was approved by the Institutional Review Board of The First Affiliated Hospital, College of Medicine, Zhejiang University. Informed consent was obtained from the patient for publication of this case report.

A 44-year-old man presented to the emergency department with severe right upper quadrant abdominal pain for 1 hour. There was no history of trauma. The onset of pain was sudden, and it was persistent in nature and worsened with movement. He denied nausea or other gastrointestinal symptoms.

Medical history included HH and gallbladder stones without any symptoms that were detected incidentally during an ultrasound examination 1 year prior. The patient reported no significant family history, alcohol intake, or smoking history.

On examination, he was found to be tachycardic (112b/min) with upper abdominal peritonism. His blood pressure was 95/63 mm Hg. Initial blood tests showed a hemoglobin of 81 g/L, white cell count of 7.86 × 10^9^/L with 84.5% segmented neutrophils (machine count), and platelet count of 155 × 10^9^/L.

**Figure 1:**
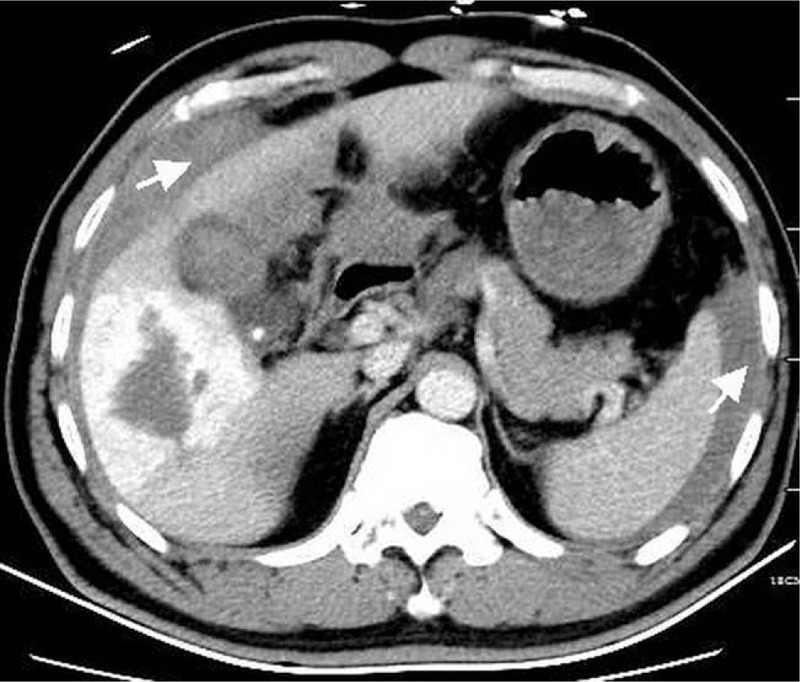
CT with intravenous contrast demonstrates a hepatic hemangioma and free high attenuation fluid around the liver and spleen (*arrow*). CT = computed tomography.

Urgent computed tomography (CT) of the abdomen with intravenous contrast demonstrated a giant HH at segment V and segment VI and free high attenuation fluid around the liver and spleen (Fig. 1). The patient was initially diagnosed with rupture and hemorrhage of the HH.

The patient was taken emergently to laparotomy, which was performed via an incision of the right mid-upper abdominal line. A large quantity of fresh and clotted blood was found around the liver and in the abdominal cavity. The fundus and body of the gallbladder completely overlaid the surface of the hemangioma. Gallbladder perforation at the fundus was identified, which caused a tear in the capsule of the hemangioma; this was the main source of active bleeding. En block resection of the hemangioma (enucleation) and gallbladder was performed using the Pringle maneuver with 12 minutes of ischemia time. The patient was transferred to the intensive care unit for 24 hours, where he received 9 units of red blood cell transfusions.

**Figure 2:**
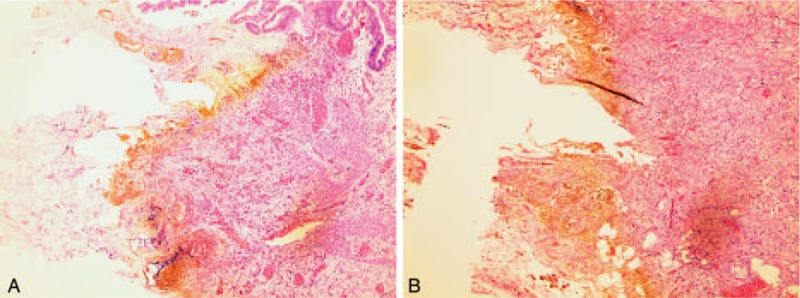
Photomicrograph shows transmural ischemic necrosis of the gallbladder extending from the mucosa (A) to the serosa (B) (H&E stain, ×50).

After transfer back to the ward, he made an uneventful recovery and was discharged home on postoperative day 8 with no complications. His postoperative histological findings revealed hepatic cavernous hemangioma and chronic cholecystitis with gallbladder perforation (Fig. 2).

## 3 Discussion

Hemangiomas are common benign tumors of the liver, generally detected incidentally during radiographic studies performed for other reasons. Based on the literature, it is clear that spontaneous rupture is a rare complication of HH. The rate of spontaneous rupture ranges from 1% to 4%,^[3]^ occurring mostly in giant HH. Pietrabissa et al^[6]^ stated that the spontaneous rupture of HH is an exceptional phenomenon. Although rare, it is associated with a high mortality rate.^[7]^ Jain et al^[8]^ reported a mortality rate ranging from 60% to 75% for spontaneous rupture with an operative mortality rate of 36.4%. Patients can die of massive hemorrhage in a short period of time. Thus it is challenging to appropriately treat HH rupture, because it is an emergent life-threatening situation. Spontaneous rupture of HH is often considered to be related to the size of the lesions.^[3]^ As the size of the hemangioma increases, so does the chance of rupture, especially if the HH is located on the surface of the liver. Yamagata et al^[9]^ concluded that lesions exceeding 10 cm in diameter may have greater internal bleeding and further growth or rupture. If the patient receives steroid therapy, the chance of rupture increases further.

Gallbladder perforation is a rare complication of gallstone disease.^[10]^ Factors contributing to spontaneous gallbladder perforation in the absence of trauma include acute cholecystitis, infection, or malignancy. The fundus is the most common site for perforation to occur. The combination of spontaneous HH rupture secondary to gallbladder perforation has not been reported in the literature, highlighting the unusual nature of this case.

**Figure 3:**
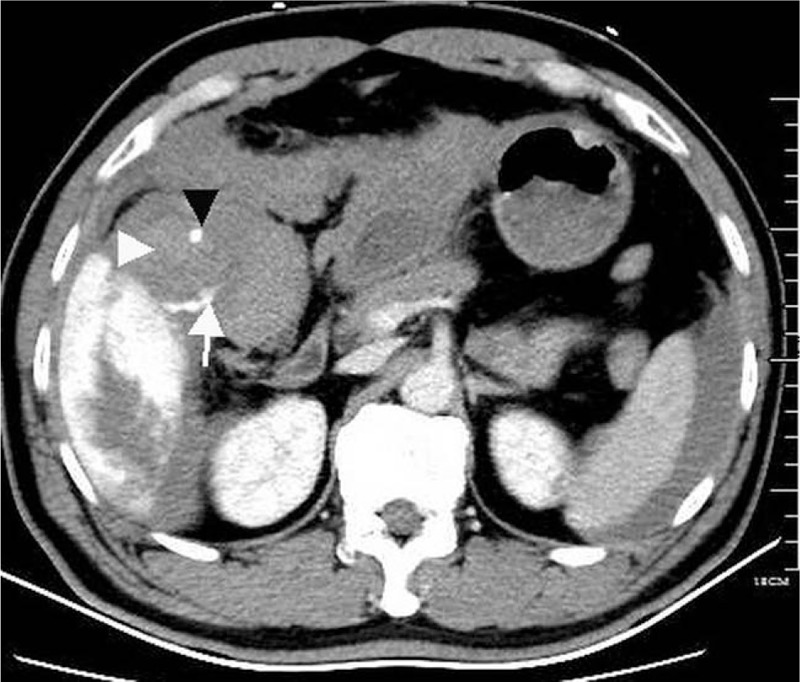
CT shows clots (*white arrowhead*) around a gallstone (*black arrowhead*) in the gallbladder and contrast extravasation from the hemangioma to the gallbladder (*white arrow*). CT = computed tomography.

Prior to laparotomy, we failed to diagnose the gallbladder perforation. After CT demonstrated a giant HH and free high attenuation fluid around the liver, spontaneous rupture of HH was quickly diagnosed. Indeed, we ignored some suspicious findings indicating gallbladder perforation that were present on the preoperative CT. Thrombi in the gallbladder and contrast extravasation from the hemangioma to the gallbladder (Fig. 3) provided clues to the diagnosis of gallbladder perforation. Because of the rarity of this complication and the absence of a history of acute cholecystitis, we missed the diagnosis.

Rupture of HH with hemoperitoneum is a serious situation and often fatal if not promptly managed; thus, surgical intervention should be considered as soon as possible.^[11]^ Ruptured HH leads to excessive hemorrhage and blood loss. Bleeding from the ruptured site is extremely difficult to stop. In case of hemodynamically unstable patients with spontaneous rupture of HH, enucleation is the preferred operation and can be performed rapidly and safely, with a potentially lower operative morbidity.^[12]^ The packing management technique for ruptured HH has a low rate of success and a high failure rate, with rebleeding and high perioperative mortality.^[13]^ Enucleation with temporary inflow occlusion (Pringle maneuver) is the first choice, and is associated with fewer postoperative complications and reduced blood loss.^[14]^ In some cases, partial resection is a safe choice that saves lives in urgent situations where HH involves large vascular structures of the liver.^[15]^ Recent studies have emphasized the role of transcatheter arterial embolization (TAE) as an effective treatment of HH.^[16]^ However, the use of TAE as an alternative to surgery in the management of ruptured HHs remains controversial.^[8,14,16]^ If the patient is stable, TAE prior to surgery may decrease intraoperative blood loss.^[2]^ In hemodynamically unstable patients with spontaneous rupture of HH, operative treatment is the preferred choice.^[12]^

Generally, giant HH (primary diameter ≥4 cm), when peripherally located and exophytic, should be considered for treatment due to its risk of rupture.^[16]^ Particular attention should be given to patients with unique clinical situations, as in the currently reported case.

In conclusion, the current case may provide additional support for treatment of HH due to the potential for spontaneous rupture. For patients with ruptured HH, enucleation with the Pringle maneuver is recommended to achieve both hemostasis and hemangioma resection.
